# The Food Contaminants Pyrrolizidine Alkaloids Disturb Bile Acid Homeostasis Structure-Dependently in the Human Hepatoma Cell Line HepaRG

**DOI:** 10.3390/foods10051114

**Published:** 2021-05-18

**Authors:** Josephin Glück, Marcus Henricsson, Albert Braeuning, Stefanie Hessel-Pras

**Affiliations:** 1 Department of Food Safety, German Federal Institute for Risk Assessment, Max-Dohrn-Straße 8-10, 10589 Berlin, Germany; josephin.glueck@bfr.bund.de (J.G.); albert.braeuning@bfr.bund.de (A.B.); 2 Wallenberg Laboratory, Department of Molecular and Clinical Medicine, Institute of Medicine, University of Gothenburg, 413 45 Gothenburg, Sweden; marcus.henricsson@wlab.gu.se

**Keywords:** pyrrolizidine alkaloids, structure dependency, hepatotoxicity, cholestasis, bile acids

## Abstract

Pyrrolizidine alkaloids (PAs) are a group of secondary plant metabolites being contained in various plant species. The consumption of contaminated food can lead to acute intoxications in humans and exert severe hepatotoxicity. The development of jaundice and elevated bile acid concentrations in blood have been reported in acute human PA intoxication, indicating a connection between PA exposure and the induction of cholestasis. Additionally, it is considered that differences in toxicity of individual PAs is based on their individual chemical structures. Therefore, we aimed to elucidate the structure-dependent disturbance of bile acid homeostasis by PAs in the human hepatoma cell line HepaRG. A set of 14 different PAs, including representatives of all major structural characteristics, namely, the four different necine bases retronecine, heliotridine, otonecine and platynecine and different grades of esterification, was analyzed in regard to the expression of genes involved in bile acid synthesis, metabolism and transport. Additionally, intra- and extracellular bile acid levels were analyzed after PA treatment. In summary, our data show significant structure-dependent effects of PAs on bile acid homeostasis. Especially PAs of diester type caused the strongest dysregulation of expression of genes associated with cholestasis and led to a strong decrease of intra- and extracellular bile acid concentrations.

## 1. Introduction

Secondary plant compounds have increasingly come into the focus of risk assessment as food contaminants in recent years. One group of these contaminants are the 1,2-unsaturated pyrrolizidine alkaloids (PAs). Humans are exposed to PAs mainly via the consumption of contaminated food. The Federal Institute for Risk Assessment identified in 2013 tea, herbal teas and honey as the main sources for human PA uptake in Western countries [1]. In addition, a consumption of contaminated salad mixes, herbs, flour or cereals can also lead to the uptake of substantial amounts of PA [2,3,4,5]. However, there are also some plants used as food that produce PA themselves such as borage. Chen et al. [6] showed that lycopsamine *N*-oxide, lycopsamine and acetyllycopsamine were the main PAs in the sample they studied. Dietary supplements based on plants containing PAs may also contribute to increased exposure to PAs. However, the total intake of PAs through their consumption cannot be estimated yet.

Although the levels of PA in most foodstuffs have been significantly reduced in recent years, exposure via highly contaminated dried herbs or herbal mixtures with PA levels of up to 3000 µg/kg seem to be possible [7].

PAs most commonly enter foods through mechanical harvesting processes, but contamination of honey by pollen from PA-containing plants or the use of PA-containing plants as herbal medicine is also a possible source of exposure [8,9,10].

Since PAs are primarily a random contamination of otherwise PA-free foods, it is not possible to predict which PAs are present in which foods. On the one hand, there are considerable differences in PA content between plant species, and on the other hand, factors such as soil conditions, climate and geographical origin can lead to considerable variations in the composition and quantity of PA within a plant species. Large intra-plant differences between different parts are also possible [11,12]. Therefore, exposure to PAs can only be estimated based on previous studies, but cannot be calculated accurately. Some studies have investigated the PA content in different foods and food supplements, and all the 1,2-unsaturated PAs used in this study were found in foodstuffs. For example, lasiocarpine and senecionine, two of the most toxic PAs examined in our study, were detected in various (herbal) teas and food supplements. Echimidine was also frequently detected [5,13].

After uptake, PAs can cause severe damage to humans and livestock after consumption of contaminated food or feed. Depending on the exposure, acute and chronic liver damage can result, such as liver hardening, ascites and the hepatic sinusoidal obstruction syndrome (HSOS), as well as liver cirrhosis, fibrosis, or liver cancer [14,15]. Due to their widespread distribution in more than 6000 plant species, especially in the *Asteraceae*, *Boraginaceae* and *Fabaceae* families, around the world, PA contamination is not locally limited [3,8,9,16,17].

**Figure 1:**
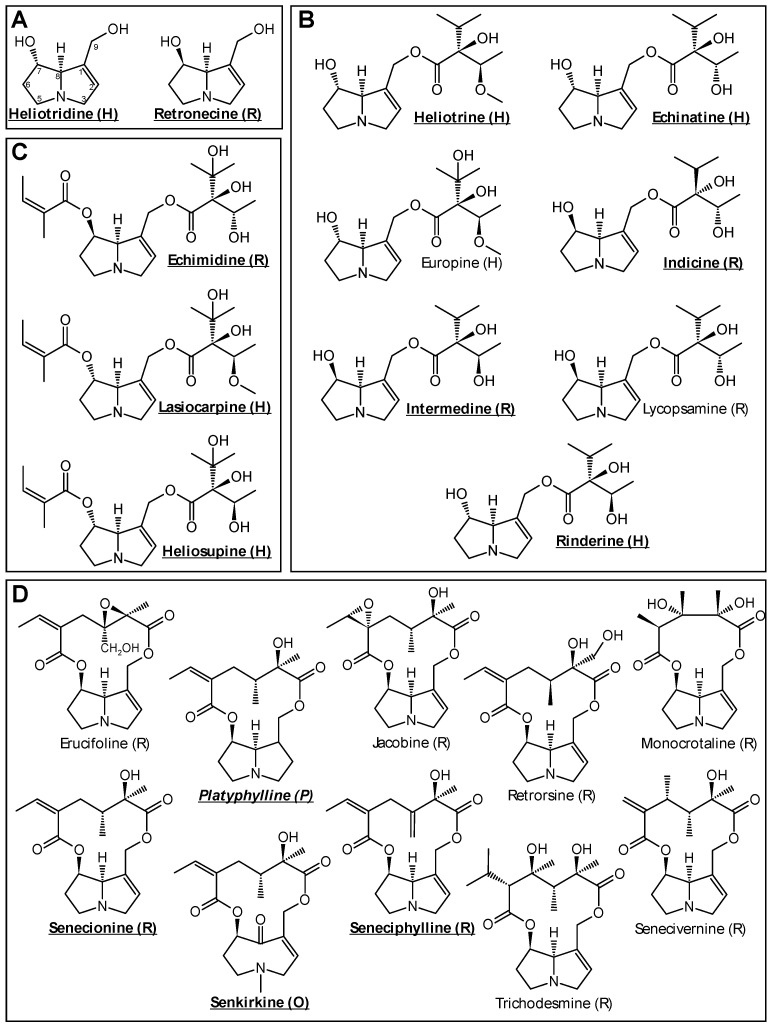
Overview of different PAs and their structural characteristics sorted by their grade of esterification: (**A**)—free bases; (**B**)—monoesters; (**C**)—open-chained diesters; (**D**)—cyclic diesters. The respective type of necine base is indicated in brackets: H—heliotrine (7S); O—otonecine (7R); P—platynecine (7R); R—retronecine (7R). The only 1,2-saturated PA, platyphylline, is indicated in italics. For cytotoxicity studies, all listed esters and the free bases heliotridine and retronecine were analyzed. The bold and underlined PAs represent the reduced test set for all subsequent experiments.

Currently, more than 660 different PAs and their corresponding N-oxides are known. About half of them are considered to exhibit genotoxic and hepatotoxic properties [18]. Chemically, PAs consist of a necine base, which can be esterified with organic acids at the OH-groups at the C 7 and C 9 positions of the double-ring system. Based on their chemical structure, PAs can be classified into different groups. PAs can be divided according to their corresponding necine base (1-hydroxymethylpyrrolizidine) into platynecine-, heliotridine-, retronecine- and otonecine-type PAs. The different grades of esterification allow a further subdivision into free bases (no esterification), monoesters and open-chained or cyclic diesters (Figure 1) [19].

The PA parent substance and its N-oxide are not very reactive as such, and therefore do not directly induce toxic effects. Due to metabolic activation in the liver, reactive metabolites such as dehydropyrrolizidine alkaloids (DHP) are formed, resulting in DNA and protein adducts [20,21,22]. For the formation of these metabolites, a double bond at the C 1/C 2 position is necessary. Therefore, the 1,2-saturated platynecine-type PAs are considered to be non-toxic [23,24].

The molecular mode of action of PAs and their metabolites in the liver is not fully understood yet. In previous studies, effects on various intracellular pathways, such as the induction of apoptosis, DNA damage response, and prostanoid synthesis were investigated [25,26,27,28,29]. In a whole-genome microarray analysis by Luckert et al. [30] in primary human hepatocytes, evidence for PA-induced disturbance of bile acid homeostasis was found. In association with PA-induced HSOS, jaundice is often diagnosed. An accumulation of bilirubin is also an indication that the normal pathway of bilirubin degradation and bile flow may be impaired [15,31].

The bile, produced in the liver, is essential for efficient absorption of fats and lipophilic substances in the intestine, as well as for the excretion of metabolites and endogenous substances from the liver. It consists of bile acids, phospholipids, cholesterol, proteins, bilirubin, electrolytes and water. The first and rate-limiting step in the de novo synthesis of bile acids is the 7α-hydroxylation of cholesterol, catalyzed by CYP7A1. The primary bile acids cholic acid (CA) and chenodeoxycholic acid (CDCA) are conjugated very rapidly with the amino acids glycine and taurine after their synthesis. Secondary bile acids are formed by conversion by microorganisms in the intestine and enter the liver after absorption from the intestine [32,33,34,35,36].

A disturbance of bile acid homeostasis can have serious consequences for the liver. Reduced bile flow can cause accumulation of potentially cytotoxic bile acids in the liver, leading to serious cell damage [37,38,39].

Some recently published studies by Waizenegger et al. [40] and Hessel-Pras et al. [41] described the disturbance of bile homeostasis by selected PAs. Due to the small number of PAs investigated in these studies, predictions on the structure–activity relationship are not applicable. Therefore, in the present study, a set of 22 structurally different PAs was systematically investigated in relation to selected endpoints associated with the disturbance of bile acid homeostasis. The endpoints in the focus of this study include the induction of cytotoxicity, changes in the expression of cholestasis-associated genes and the influence on the levels of intra- and extracellular bile acids in the metabolically competent human hepatoma cell line HepaRG.

## 2. Materials and Methods

### 2.1. Chemicals

The PAs used in this study, except platyphylline, were purchased from Phytoplan Diehm & Neuberger GmbH (Heidelberg, Germany) with a purity of at least 95%. Platyphylline was obtained from BOC Sciences (New York, NY, USA). All PAs were dissolved in 50% acetonitrile (ACN, Sigma-Aldrich, Taufkirchen, Germany)/50% H_2_O. The 5 mM stock solutions were stored at −20 °C. All other chemicals used in this study were obtained from Merck (Darmstadt, Germany) or Sigma-Aldrich (Taufkirchen, Germany) in the highest available purity.

### 2.2. HepaRG Cell Culture

The human hepatoma cell line HepaRG was purchased from Biopredic International (Saint-Gregoire, France). The cells were cultivated for two weeks in proliferation medium composed of William’s Medium E with stable glutamine (PAN Biotech, Aidenbach, Germany), 10% fetal bovine serum (FBS Good forte, PAN Biotech, Aidenbach, Germany), 100 U/mL penicillin and 100 µg/mL streptomycin (Capricorn Scientific, Ebsdorfergrund, Germany), 5 µg/mL human insulin (PAN Biotech, Aidenbach, Germany) and 50 µM hydrocortisone hemisuccinate (Sigma-Aldrich, Taufkirchen, Germany). After the proliferation phase, differentiation was initiated by adding 1.7% dimethyl sulfoxide (DMSO, Merck, Darmstadt, Germany). The differentiation of HepaRG cells was completed after two weeks of cultivation in differentiation medium consisting of proliferation medium with 1.7% DMSO. All experiments with HepaRG were conducted with differentiated cells seeded at passages 16 to 20.

### 2.3. Cell Viability Assay

For cytotoxicity testing, 9000 HepaRG cells per well were seeded in the inner 60 wells of a 96-well plate. After proliferation and differentiation, FBS was set to 2% for 48 h before incubation. DMSO concentration of 1.7% was kept to reach the maximum expression of various CYP enzymes and transporters to ensure a strong metabolic activation of PAs [42]. The cells were treated with PAs in different concentrations as indicated in the figures. After 24 h of incubation, 10 µL of undiluted MTT reagent (3-(4,5-dimethylthiazol-2-yl)-2,5-diphenyltetrazolium bromide, Sigma-Aldrich, Taufkirchen, Germany) were added per well. After an incubation time of 30 min at 37 °C, the supernatant was removed and the formazan crystals were dissolved in 130 µL isopropanol with 0.7% sodium dodecyl sulfate (SDS, Merck, Darmstadt, Germany) for approximately 30 min under shaking and light protection. Absorption was measured at 570 nm, with 630 nm as reference wavelength [43]. Cell viability was calculated by subtracting the background and normalizing the treatment to solvent control. Solvent control was set to 100%. Four independent experiments with three replicates each were performed.

### 2.4. Isolation of Total RNA and Quantitative Real-Time Polymerase Chain Reaction (qPCR)

The effect of PAs on the expression of cholestasis-associated genes was analyzed using real-time qPCR. HepaRG cells were seeded at a density of 0.2 × 10^6^ cells per well in 6-well plates. After proliferation and differentiation, DMSO and FBS were set to 0.5% and 2% for 48 h before incubation to ensure inducibility of gene expression. The cells were treated with PAs in different concentrations as indicated in the figures. After 24 h of incubation, the cells were washed two times with ice-cold phosphate-buffered saline (PBS). The total RNA was extracted using the RNeasy Mini Kit (Qiagen, Hilden, Germany) following the manufacturer’s protocol, including the on-column DNase digestion step. Concentration and purity of the RNA was measured at 260 and 280 nm by a TecanM200Pro using a NanoQuant plate.

cDNA was synthesized by transcribing 1 µg of RNA, using the High Capacity cDNA Reverse Transcriptase Kit according the manufacturer’s instructions (Applied Biosystems, Foster City, CA, USA). qPCR was conducted with Maxima SYBR Green/ROX qPCR Master Mix (Thermo Fisher Scientific, Waltham, MA, USA), 300 nM primers and 1 µL cDNA per sample in a total volume of 10 µL. The amplification protocol comprised the following steps:

- initial denaturation (15 min at 95 °C)
- 40 cycles of denaturation (30 s at 95 °C) and annealing/elongation (1 min at 60 °C)
- final elongation (10 min at 60 °C)
- dissociation curve

All qPCRs were performed on a 7900HT Fast Real-Time PCR System (Applied Biosystems, Foster City, CA, USA) using the 384-well format. Primer sequences used for the amplification are summarized in Table S2 in the supplementary material. The primers were obtained from Eurofins (Hamburg, Germany). The results were evaluated according to the 2^–ΔΔCt^ method [44], normalized to the housekeeping gene GUSB (β-glucuronidase) and referred to the solvent control. Three replicates per sample were measured.

### 2.5. Bile Acid Quantification

The content of different bile acids was detected via UPLC-MS/MS in the cell culture supernatant and cell lysates. Thus, HepaRG cells were seeded at a density of 0.2 × 10^6^ cells per well in 6-well plates. After the normal proliferation and differentiation period of four weeks, the FBS level in the medium was set to 2% for 48 h. DMSO concentration of 1.7% was kept to reach the maximum expression of various CYP enzymes and transporters to ensure a strong metabolic activation of PAs [42]. Before the incubation with PAs, the cells were washed once with PBS (room temperature) to remove the traces of FBS from cell culture medium. The incubation with PAs was conducted with FBS-free differentiation medium to reduce the background signal from bile acids contained in FBS, as described in the study by Sharanek et al. [39]. The volume of incubation medium (differentiation medium containing the respective amount of PAs) was reduced by 50% to 1 mL per well, to increase the concentration of excreted bile acids in the supernatant. Cells were incubated in triplicates to pool the medium and cells of three wells during harvesting, in order to facilitate analytical determination of bile acids present only at low levels. After incubation for 48 h, the medium was collected and stored at −80 °C. Cells were washed twice with PBS, trypsinized for 15 min at 37 °C and collected (Trypsin-EDTA, Capricorn Scientific, Ebsdorfergrund, Germany). After a second washing step with 1 mL PBS and centrifugation at 250× *g* for 5 min, the supernatant was discarded and the cell pellets were stored at −80 °C.

Bile acid analysis was conducted using UPLC coupled to tandem mass spectrometry as described previously [45]. Briefly, bile acids in cell lysates and medium were extracted by adding methanol containing deuterated internal standards. After vortexing and centrifugation, the methanol was evaporated under a stream of nitrogen, the samples were reconstituted in methanol:water [1:1 *v*/*v*] and injected onto the UPLC system (Infinity1290, Agilent Technologies, Palo Alto, CA, USA). Separation was made on a Kinetex C18 column (Phenomenex, Torrance, CA, USA) using H_2_O and ACN as mobile phases. Detection was made in negative mode using a QTRAP 5500 mass spectrometer (Sciex, Concord, Canada). Three independent experiments were performed.

### 2.6. Statistical Analysis

For statistical analysis, SigmaPlot 14.0 software (Systat Software, Erkrath, Germany) was used. Statistically significant differences in a concentration series were calculated using a one-way ANOVA. Following the differences of the treated samples versus the respective solvent control were tested using Dunnett’s post hoc analysis. Differences that were statistically significant are indicated by * *p* < 0.05, ** *p* < 0.01, *** *p* < 0.001.

## 3. Results

### 3.1. PA-Induced Cytotoxic Effects in HepaRG Cells

To elucidate the structure-dependent effects of PAs on HepaRG cells and to establish a suitable concentration range for further investigations, cell viability studies were performed. Metabolically competent HepaRG cells were treated with the 22 PAs for 24 h at six different concentrations ranging from 0.1 to 250 µM. The upper concentration limit resulted from the low PA solubility and the resulting high concentration of solvent in the incubation medium (max. 2.5% ACN). The high concentration of 1.7% DMSO in the medium was chosen to obtain the highest possible activity of xenobiotic-metabolizing CYP enzymes for effective bioactivation of PAs [42].

**Figure 2:**
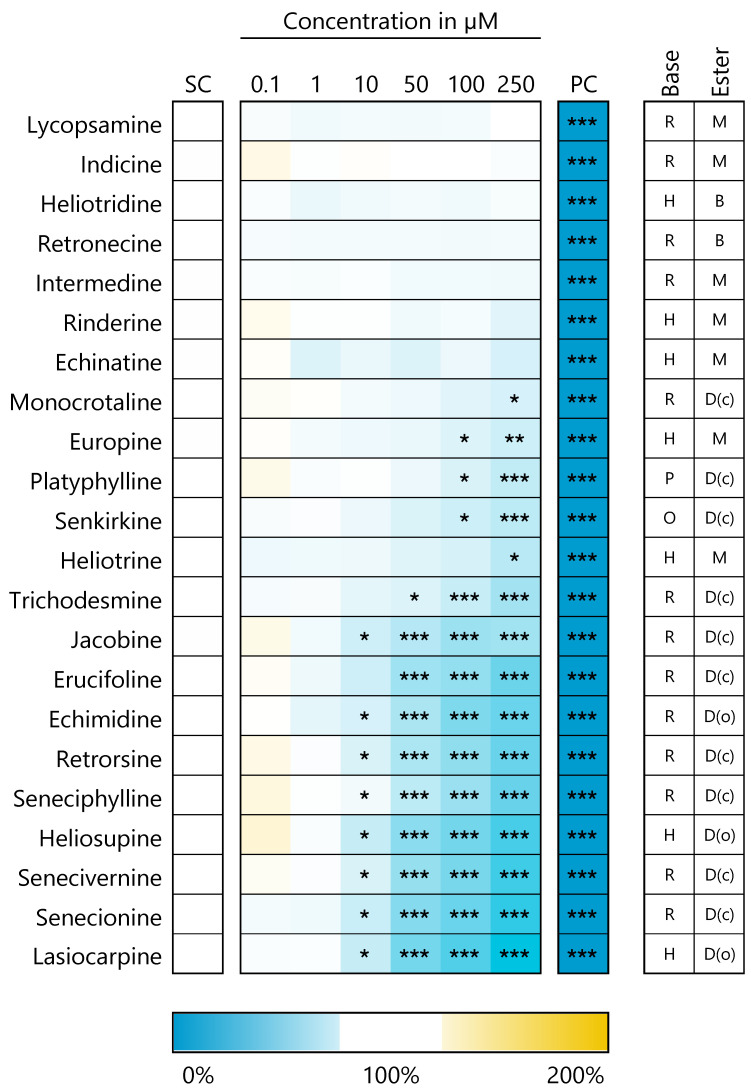
Decreasing viability of HepaRG cells 24 h after PA exposure. Cell viability was measured by the MTT assay. The mean values out of three biological replicates with three technical replicates each were normalized to the solvent control (SC, 2.5% ACN, 1.7% DMSO). Triton X-100 (0.05%) was used as positive control (PC). The heat map shows the cell viabilities in percent of the solvent control. Blue color indicates a decrease in cell viability and yellow indicates an increase. Statistics: * *p* < 0.05, ** *p* < 0.01, *** *p* < 0.001 (one-way ANOVA followed by Dunnett‘s post hoc test versus the solvent control). Mean values, standard deviations and *p*-values can be found in the supplemental material. Abbreviations for structural characteristics of the PAs: retronecine (R), heliotrine (H), otonecine (O) or platynecine (P) type; free base (B), monoester (M), open-chained diester (D(o)) or cyclic diester (D(c)).

The results of the MTT assay are summarized in the heat map in Figure 2. For better overview, the PAs were sorted in descending order according to cell viability detected at the highest concentration. Clear differences in cell viability became obvious at a concentration of 250 µM, varying between 100 and 41%. Upon closer examination, it can be seen that among the less cytotoxic PAs (cell viability > 80% at 250 µM), the free bases heliotridine and retronecine, as well as monoesters, such as lycopsamine, indicine, intermdine, rinderine, echinatine and europine, were mainly represented. Moderate (cell viability between 80 and 60%) to strong toxicity (cell viability < 60%) was more likely to be induced by open-chained and cyclic diesters. Platyphylline, which is actually considered to be non-toxic, nevertheless led to a statistically significant reduced cell viability of 88% and 81% at the highest concentrations of 100 µM and 250 µM, respectively. Corresponding cytotoxicity data for HepaRG cells with a reduced DMSO content of only 0.5% have been published previously [25]. The levels of the effects here turned out to be somewhat smaller, but the order of PAs by strength of induced cytotoxicity is comparable.

### 3.2. PAs Affect Structure-Dependently Expression of Genes Involved in Bile Acid Homeostasis

Effects of PAs on the regulation of cholestasis-associated gene expression were elucidated with a test set of 14 selected PAs in which representatives of all structural groups were present. qPCR was used to investigate the expression of 45 genes associated with cholesterol and bile acid metabolism. HepaRG cells were treated with PAs at concentrations of 5, 21, and 35 µM for 24 h. These concentrations were chosen to induce no or only weak cytotoxic effects. For the analysis of gene expression, the DMSO concentration was lowered from 1.7% to 0.5% to ensure inducibility of gene expression.

**Figure 3:**
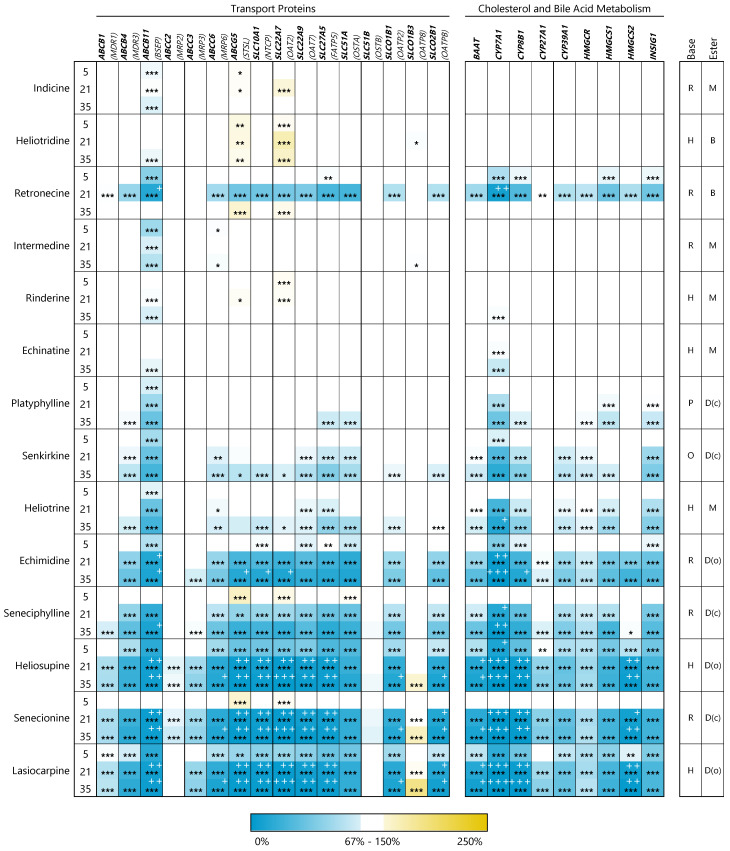

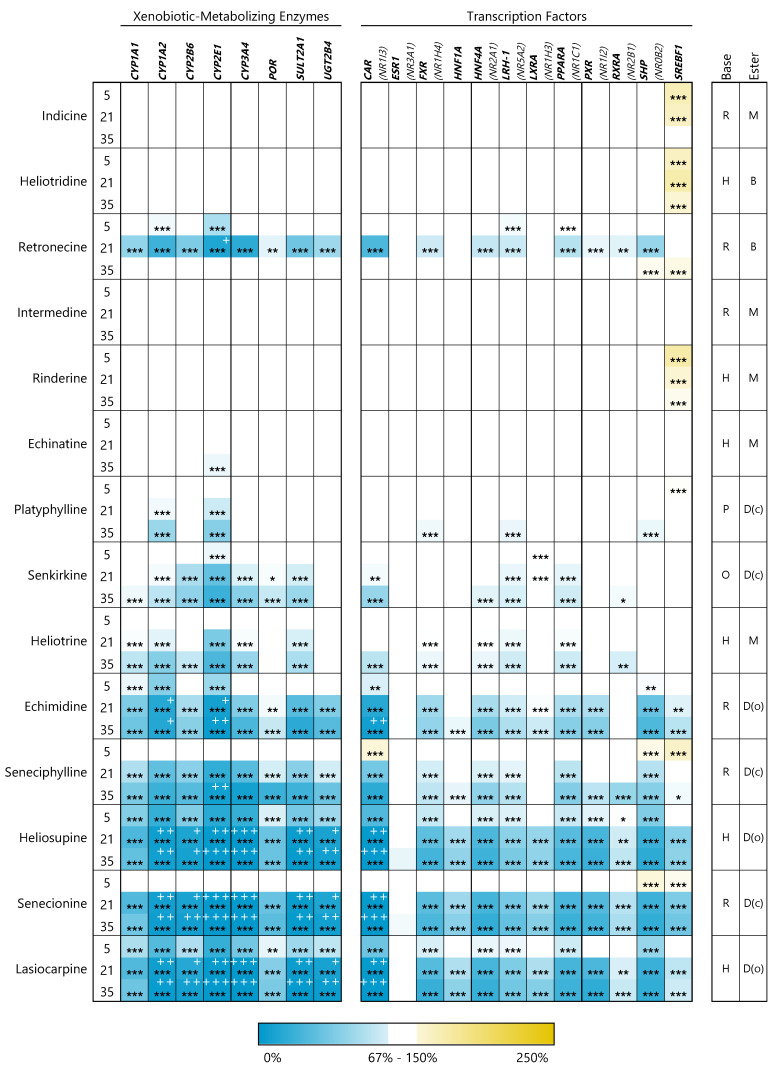
Changes in expression of cholestasis-associated genes after PA treatment of HepaRG cells for 24 h. Differentiated HepaRG were treated with PAs in concentrations of 5, 21 and 35 µM. The Ct-values were evaluated according to the 2^-ΔΔCt^ method by normalizing Ct-values of the respective gene to the housekeeping gene β-glucuronidase (GUSB) and by referring to solvent-treated cells (0.35% ACN and 0.5% DMSO). The cutoff for gene expression regulation was set from 67% to 150% of solvent control. Changes in gene expression within this range were considered not to be biologically relevant. The cells of the heat map show the changes in gene expression of the target genes in percent of the solvent control as means of three replicates. Blue color indicates a downregulation and yellow color an upregulation of gene expression. Expression levels below 10% of solvent control are additionally highlighted by white + (+ expression level below 10%; ++ expression level below 5%; +++ expression level below 1%). Statistics: * *p* < 0.05, ** *p* < 0.01, *** *p* < 0.001 (one-way ANOVA followed by Dunnett‘s post hoc analysis versus the respective solvent control). Mean values, standard deviations and *p*-values are summarized in the supplemental material (Table S3), as well as the sequences of used primers and the full-length names and synonyms of the detected genes (Table S2). Structural characteristics: retronecine (R), heliotrine (H), otonecine (O) or platynecine (P) type; free base (B), monoester (M), open-chained diester (D(o)) or cyclic diester (D(c)).

In Figure 3 the induction of gene expression in percent of the solvent control is represented as the heat map. Further details such as means and standard deviations are summarized in the supplemental material (Table S3). For better comparability of the results for the different endpoints tested, the PAs were always sorted according to their cytotoxicity-inducing potential, as shown in Figure 2. The analyzed genes were classified related to the function of their corresponding proteins into the groups transport proteins, xenobiotic-metabolizing enzymes, transcription factors, and enzymes of cholesterol metabolism.

In the heat map, it can be clearly seen that the expression of all investigated genes was downregulated or unchanged without an exception. A significant upregulation of expression was not detected for any of the examined genes. In addition, it is noticeable that the effects, particularly in the case of the xenobiotic-metabolizing enzymes, were sometimes extremely strong with a reduction of the expression down to around 0.02% (*CYP7A1*, senecionine, 35 µM). Summarizing the gene expression data, it can additionally be concluded that PAs showing the strongest effects on cell viability also have more pronounced effects on the regulation of gene expression.

#### 3.2.1. Transport Proteins

Within the group of transporters, the bile salt export pump (BSEP, ABCB11), the Na+/taurochlorate cotransporting polypeptide (NTCP, SLC10A1) and the bile acyl-CoA synthetase (SLC27A5) interact very specifically with bile acids and are thus directly involved in bile acid and cholesterol homeostasis. For these transporters, a very significant concentration-dependent downregulation of gene expression was observed after treatment with the three most cytotoxic PAs (lasiocarpine, senecionine, and heliosupine). Even after exposure of HepaRG cells to echimidine and seneciphylline, a weaker but still significant downregulation was evident at 21 and 35 µM of the PAs. Less or non-cytotoxic PAs exhibited no or only weak effects on the expression of genes encoding the abovementioned transporters. A similar pattern was observed for the expression of the less bile-acid-specific transport proteins ABCB4, ABCC3, ABCC6, ABCG5, SLC22A7, SLC22A9, SLC51A, SLCO1B1 and SLCO2B1. For the genes encoding the transport proteins ABCB1, ABCC2, SLC51B, SLCO1B3, no substantial changes in gene expression were detected.

#### 3.2.2. Enzymes of Cholesterol and Bile Acid Metabolism

While the expression of the genes for the enzymes of HMG-CoA synthesis (HMGCS1/2) was significantly downregulated, no change was detected in the gene expression of the rate-limiting HMGCR. The expression of INSIG1, responsible for the regulation of HMGCR expression via negative feedback was slightly downregulated (maximum reduction to 16% for 21 µM senecionine). BAAT, an enzyme for the amidation of bile acids with taurine and glycine, showed a significant reduced gene expression down to 5.5% of the solvent control. The decrease of the gene expression levels was stronger, the more cytotoxic and higher concentrated the respective PA was. The transcripts of CYP enzymes directly involved in the catabolism of cholesterol or the de novo synthesis of bile acids (CYP7A1, CYP8B1) were strongly downregulated in a structure-dependent manner, while the expression of *CYP27A1* and *CYP39A1*, relevant for the synthesis of secondary bile acids, were not affected at all.

#### 3.2.3. Xenobiotic-Metabolizing Enzymes

The expression levels of all investigated genes of xenobiotic-metabolizing enzymes were affected in a structure- and concentration-dependent manner. For *CYP1A1* and *POR*, however, the decrease of mRNA content was rather weak. For these genes we could still detect around 20–30% of the transcript level of the solvent control after the exposure with the strongest regulating PAs. The transcription of the genes encoding the enzymes CYP1A2, CYP2B6, CYP2E1, CYP3A4, SULT2A1 and UGT2B4 was strongly downregulated.

#### 3.2.4. Transcription Factors

In general, gene expression of transcription factors was less affected by PA treatment in HepaRG cells. For transcription factors typically expressed in the liver (*FXR*, *HNF1A*, *HNF4A*, *LRH-1*, *LXR*, *PPARA*, and *PXR*), a downregulation of expression at the mRNA level down to 14% compared to untreated cells could be detected. No changes were detected for the nuclear receptors *ESR1* and *RXRA*. The gene expression of the constitutive androstane receptor (CAR) was strongly affected by PAs in a structure-dependent manner: a reduction of the CAR transcript down to 0.6% was detected in the treated HepaRG cells for the most cytotoxic PAs senecionine, heliosupine and lasiocarpine.

### 3.3. PAs Disturb Bile Acid Homeostasis Intra- and Extracellular

The amounts of the primary bile acids cholic acid (CA) and chenodeoxycholic acid (CDCA), as well as the respective glycine- or taurine-conjugated bile acids (GCA, TCA, GCDCA, TCDCA), were measured in the cell lysates and medium supernatants of PA-treated HepaRG cells by UPLC/MS after 48 h of incubation with PAs under serum-free conditions. Due to their formation by gut bacteria, secondary bile acids were not considered in our model.

**Figure 4:**
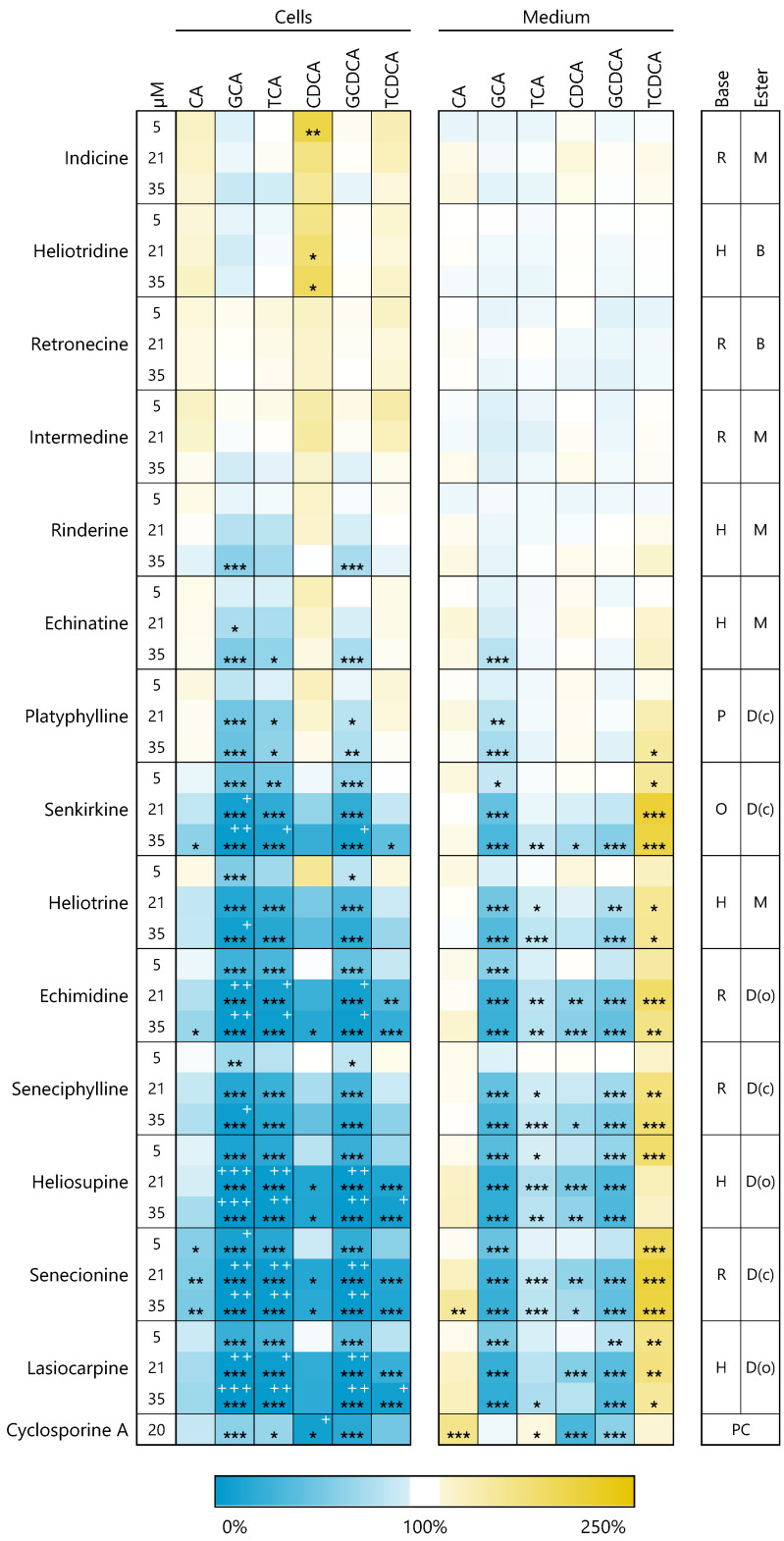
Changes in the intra- and extracellular bile acid concentration after PA treatment for 48 h. Differentiated HepaRG cells were treated with PAs in concentrations of 5, 21 and 35 µM under serum-free conditions. The cells of the heat map are colored according to the relative change of the respective bile acid as mean of three replicates compared to untreated cells (solvent control, 0.35% ACN and 1.7% DMSO). An increase is indicated by yellow color and blue filling indicates a decrease compared to the solvent control (100%). Cyclosporine A (20 µM) was used as positive control (PC) for the induction of cholestasis [46]. Bile acid levels below 10% of solvent control are additionally highlighted by white + (+ below 10%; ++ below 5%; +++ below 1%). Statistics: * *p* < 0.05, ** *p* < 0.01, *** *p* < 0.001 (one-way ANOVA followed by Dunnett‘s post hoc analysis versus the respective solvent control). Mean values, standard deviations and *p*-values are summarized in the supplemental material (Table S4). Structural characteristics: retronecine (R), heliotrine (H), otonecine (O) or platynecine (P) type; free base (B), monoester (M), open-chained diester (D(o)) or cyclic diester (D(c)). Abbreviations for measured bile acids: cholic acid (CA), glycocholic acid (GCA), taurocholic acid (TCA), chenodeoxycholic acid (CDCA), glycochenodeoxycholic acid (GCDCA), taurochenodeoxycholic acid (TCDCA).

The detected intra- and extracellular amounts of bile acids are summarized in Figure 4. PAs were sorted according to their potential to induce cytotoxicity in HepaRG cells (cp. Figure 2). The amounts of bile acids in the medium of the treated cells were normalized to the values obtained with medium of the respective solvent control (untreated cells, set to 100%). The amount of bile acids in the cell lysates was first normalized to the total protein content of the cells and then to the corresponding solvent control.

The pattern of the effects on bile acid homeostasis is obviously similar to the effects on gene regulation and cytotoxicity. The higher the cytotoxic potential of the PA, the stronger the effects on bile acid balance. Furthermore, the amount of bile acids in the medium and in the cells decreased significantly with increasing concentration of the respective PA. The effect was much more pronounced in the cell lysates than in the medium. Besides the reduced amounts of most detected bile acids, the conjugated bile acid TCDCA represents an exception due to a slightly increased level in the medium of PA-treated cells. Additionally, a slight but significant increase of intracellular CDCA could be detected after treatment with the non-cytotoxic PAs indicine and heliotridine.

Cyclosporine A, a drug known to induce cholestasis, was used as a positive control [46]. Exposure of HepaRG cells to 20 µM cyclosporine A for 48 h showed similar but much weaker effects than treatment with toxic PAs.

## 4. Discussion

Due to their widespread distribution and their strong hepatotoxic properties, 1,2-unsaturated PAs are among the most important naturally occurring toxins. In current risk assessment, the general assumption that all PAs have equipotent hepatotoxic properties regardless of their structural characteristics has been followed [13]. However, many recent studies suggest structure-dependent effects of PAs on various endpoints like the induction of cytotoxicity and apoptosis, the occurrence of DNA double-strand breaks, and the formation of micronuclei and DHP-DNA adducts [26,47,48,49,50,51,52]. For all studies mentioned, a highly comparable order of PAs, sorted by their respective effect level, could be observed. The group of PAs showing the strongest effects, if used in the respective test set, always included the representatives of the open-chained diesters of the heliotridine type (lasiocarpine and heliosupine), and the cyclic diesters of the retronecine type (senecionine and seneciphylline). Echimidine (open-chained diester, retronecine type) also always showed a clear effect on the endpoint studied, despite some variations. This ranking has been observed independently of the test system or endpoint investigated and is comparable to the observations in this study. As discussed in detail in Glück et al. [25], it is becoming increasingly apparent that the representatives of the open-chained and cyclic diester groups of the heliotrine and retronecine type show the strongest toxic effects, whereas free bases and monoesters show no or only weak effects. This pattern of effect levels was also observed in the present study dealing with the disturbance of bile acid homeostasis.

A balanced bile acid level is important for normal liver function. Changes in the concentration of cytotoxic bile acids in the liver can lead to severe damage to hepatocytes. Jaundice and HSOS are frequently reported symptoms after PA intoxication, and indicate a connection between PA exposure and disturbance in bile flow [31]. Currently, there are very few studies dealing with an association between PA exposure and bile acid or cholesterol imbalance. Yan and Huxtable identified the first evidence for such a relation in the 1990s by studying changes in bile flow and bile composition in rat liver after PA administration [53,54]. They showed that detoxification of PAs occurs via glutathione conjugation. The resulting hydrophilic conjugates are then efficiently secreted via the bile. Taurine from liver cells and bile also appears to reduce PA toxicity [55]. Furthermore, Yan and Huxtable [56] found evidence that different PAs can induce different effects or effect levels, as retrorsine and senecionine stimulated bile flow and monocrotaline and trichodesmine did not.

Xiong et al. [57,58] investigated the effect of the PA senecionine as a single substance, or of extracts from PA-containing plants, on the expression of genes associated with the synthesis and transport of bile acids in two in vivo studies. In addition, the concentration of various bile acids was measured in the serum of rats after PA ingestion. In both studies, an increase in serum concentration was detected for all bile acids analyzed. In addition to the abovementioned studies, Hessel-Pras et al. [41] showed an induction of liver necrosis, inflammation and a disturbance of bile acid homeostasis in mouse liver after the exposure to senecionine, resulting in increased bile acid concentrations in serum.

Effects on bile acid homeostasis have also been described in several in vitro studies. Comparable to the information provided by the abovementioned in vivo studies, Luckert et al. [30] found indirect evidence for effects of PAs on the metabolism and transport of bile acids in primary human hepatocytes. After treatment with four different PAs, downregulation of the transcript level was observed for the liver transport proteins ABCB11 (BSEP), ABCC2, ABCC3, ABCC6, SLC10A1 (NTCP), SLC22A7, SLC22A9, SLCO1B1, SLCO1B3, SLCO2B1. Waizenegger et al. [40] examined the regulation of an extensive set of genes associated with bile acid homeostasis, with an experimental setup comparable to the design of this study. After treatment of HepaRG with four different PAs, the qPCR analyses and bile acid content measurements showed results very similar to the present study, including a strong reduction of gene expression of several enzymes involved in bile acid uptake, synthesis, metabolism, and excretion. Enzymes involved in de novo bile acid synthesis (CYP7A1, CYP8B1, BAAT) show decreased transcript levels in the in vitro experiment of this study. In rat liver, however, this change is only partially visible and rather weak. After exposure to senecionine, the expression of *Cyp7a1* and *Baat* was slightly decreased, whereas after ingestion of *Senecio vulgaris* extract, the transcript levels of *Cyp8b1* and *Baat* were reduced [57,58].

According to formerly published results from Waizenegger et al. [40], the analysis of the intra- and extracellular bile acid levels in the present study shows a strong reduction of their concentrations. The effects of the cholestasis-inducing drug cyclosporine A [46] were very similar to the PA-induced variations in bile acid balance, indicating a possible cholestasis-inducing potential of PAs in vivo. Overall, the changes of bile acid amounts were intracellularly stronger than extracellularly. This may be due to the fact that the cells are able to secrete the toxic bile acids very efficiently. The concentration changes for the primary bile acids CA and CDCA were much weaker than for their corresponding conjugates. However, since the unconjugated bile acids are rapidly converted into the glycine and taurine conjugates, their intracellular levels and effects on overall bile acid concentrations are very small compared to the conjugated bile acids.

Comparing gene expression data from the two in vivo studies in rat liver and the in vitro study in human HepaRG, many similarities can be found [40,57,58]. The genes of the nuclear receptors FXR and SHP are less expressed in both, in vitro in the human cell system and in vivo in the rat liver, after exposure to senecionine and the extract of *Senecio vulgaris*. The gene expression of the transporters SLC10A1 (NTCP) and SLCO1B1 is also significantly downregulated in all three studies. These transporters are responsible for the uptake of bile acids into hepatocytes from the blood [59,60,61,62]. Reduced expression of these transporters could result in lower bile acid concentrations in hepatocytes and thus counteract cholestasis. Differences are apparent in the regulation of the transporters ABCB11 (BSEP), SLC22A7, and ABCC3. While *Abcb11* (*Bsep*) and *Slc22a7* showed hardly any altered expression at the mRNA level in rat liver, their orthologs were significantly downregulated in human HepaRG cells after PA exposure. ABCB11 (BSEP) transports bile acids from the hepatocytes into the bile, whereas SLC22A7 imports organic anions (potentially bile acids) from the basolateral (blood) side into the hepatocytes [63]. *ABCC3*, on the other hand, showed only minimal downregulation in cell culture experiments, whereas gene expression was greatly increased in the rat. ABCC3 is responsible for exporting bile acids to the basolateral side of hepatocytes. This pathway is considered as an alternative route for the secretion of bile acids from cholestatic hepatocytes [64]. Thus, *Abcc3* upregulation in vivo fits very well with the increased serum concentration of bile acids that was also measured. However, this discrepancy of enhanced bile acid amounts in vivo to low levels observed in our in vitro model can probably be associated with the simplified 2D culture of HepaRG cells. Despite differentiation of HepaRG into hepatocyte-like and biliary epithelium-like cells, the culture model does not fully reflect the compartmentalization and polarization of the liver that may strongly affect the regulation of various processes like the efflux of bile acids. Nevertheless, the results of the cell culture experiment clearly show a PA-mediated impairment of bile acid homeostasis in human hepatocyte-like HepaRG cells.

## 5. Conclusions

In the present study, we have shown that PAs have a significant structure-dependent effect on bile acid homeostasis. We were able to demonstrate that especially PAs of the diester type caused strongest dysregulation of expression of several genes responsible for bile acid synthesis, uptake and secretion in HepaRG cells. Furthermore, the amounts of intra- as well as extracellular bile acids were strongly affected. The dramatic decreases of intra- and extracellular bile acid amounts were also predominantly detected for diester-type PAs showing a clear impairment of the bile acid balance, which may contribute to cholestatic liver disease in vivo. Therefore, our in vitro results support in vivo observations [36,53,54] that PAs could stimulate the formation of cholestasis. Our data show very impressively the structure dependence of this effect, although this correlation must be verified and further investigated in vivo.

Nevertheless, for a reliable risk assessment of PAs, some knowledge gaps need to be filled. Especially with regard to the classification of PAs according to their structural characteristics and the resulting differences in their toxicity, further refining studies are necessary. Important aspects for future studies should be the analysis of toxicokinetics and the associated metabolic reactions of toxification and detoxification. Further in vivo and in vitro studies are therefore essential for a more precise and reliable assessment of the risk to human health from PA contamination in food.

## Data Availability

The data presented in this study are available in the Supplementary Materials.

## Supplementary Materials

The following are available online at https://www.mdpi.com/article/10.3390/foods10051114/s1, Table S1: Cytotoxicity MTT, Table S2: PCR Primer Sequences, Table S3: Gene expression PCR, Table S4: Bile acid quant. LC-MS.

## Abbreviations

ACN	Acetonitrile
BA	Bile acids
CA	Cholic acid
CDCA	Chenodeoxycholic acid
CYP	Cytochrome P450
DHP	Dehydropyrrolizidine alkaloid
DMSO	Dimethyl sulfoxide
FBS	Fetal bovine serum
GCA	Glycocholic acid
GCDCAHSOS	Glycochenodeoxycholic acidHepatic sinusoidal obstruction syndrome
MTT	3-(4,5-Dimethylthiazol-2-yl)-2,5-diphenyltetrazoliumbromid
PA(s)	Pyrrolizidine alkaloid(s)
PBS	Phosphate-buffered saline
qPCR	quantitative Polymerase chain reaction
SDS	Sodium dodecyl sulfate
TCA	Taurocholic acid
TCDCA	Taurochenodeoxycholic acid
